# The Cause of Bovine Tuberculosis

**Published:** 1882-07

**Authors:** 


A PARASITE THE CAUSE OF BOVINE TUBERCULOSIS.—Dr. Robert Koch, a German physician, has discovered a minute organism in the lungs of consumptive animals. He considers it to be the veritable cause of tuberculosis in all its forms in man as well as in beast. The organism in question is rod-shaped, and about one six-thousandth of an inch long. It is found in tuberculous matter everywhere, even in tuberculous sputa. Dr. Koch cultivated it in ox-serum, then inoculated animals with the cultures. In this way he claims that he has produced a true tuberculosis. The discoverer of the new organism thinks that bovine and human tuberculosis are identical; also that tuberculosis can be given to man by the milk, and perhaps flesh, of tuberculous cows. Dr. Koch's conclusions have a very important bearing on veterinary sanitary science, if they are true. This is far from being proven as yet.

